# Supplementary material for: In Vitro Evaluation of the PMN Reaction on a Collagen-Based Purified Reconstituted Bilayer Matrix (PRBM) Using the Autologous Blood Concentrate PRF
Source: Biomedicines. 2025 May 20;13(5):1239. doi: 10.3390/biomedicines13051239 (PMC12109348; doi:10.3390/biomedicines13051239)
Supplement: Supplementary file 1 [file biomedicines-13-01239-s001.zip › biomedicines-3615039-supplementary.pdf]

A

CYTOKINE	D1	D2	D3	D4	D5	D6	D7	D8	D9	MW	SD
**ELA**	619,63	953,69	1020,63	1246,03	1240,86	1260,59	1120,24	957,57	873,17	1032,49	211,11
**TGF**	287,21	300,78	282,52	344,85	357,06	357,45	381,31	370,91	395,85	341,99	41,82
**IL10**	64,24	67,86	61,51	55,18	55,04	53,92	59,51	63,74	64,31	60,59	4,9
**IL4**	91,95	91,67	91,19	91,50	90,37	91,92	88,14	91,09	89,23	90,78	1,31
**VEGF**	18,62	18,45	18,09	18,22	17,97	17,96	12,84	14,81	16,72	17,07	1,98
**TNF**	278,99	275,77	281,20	169,75	167,35	167,14	110,98	142,23	130,12	191,50	68,12
**IL15**	26,96	26,55	26,54	26,76	27,14	27,41	28,52	27,11	26,62	27,06	0,62
**IL1**	210,92	224,50	224,71	45,89	44,72	43,55	5,18	8,20	4,24	90,21	98,90
**IL6**	885,22	954,83	917,92	951,11	974,53	990,00	875,02	853,31	812,09	912,67	60,10

B

CYTOKINE	D1	D2	D3	D4	D5	D6	D7	D8	D9	MW	SD
**ELA**	4087,02	3703,80	3686,98	4079,58	4202,79	4206,02	2421,23	2361,41	2373,37	3458,02	826,35
**TGF**	290,98	292,35	277,04	303,54	302,92	312,87	458,20	449,44	351,28	337,62	69,01
**IL10**	77,58	76,69	103,77	77,80	95,57	80,27	73,36	68,74	66,85	80,07	12,10
**IL4**	89,63	90,18	89,48	88,23	89,12	90,97	91,24	90,23	91,98	90,11	1,15
**VEGF**	19,41	19,26	19,09	17,14	17,10	17,14	15,82	15,82	15,81	17,39	1,50
**TNF**	349,86	334,19	324,32	242,44	243,83	247,38	3,42	4,35	2,48	194,69	148,98
**IL15**	25,72	25,19	25,43	25,46	25,70	25,53	25,50	25,64	25,28	25,49	0,18
**IL1**	161,21	193,14	197,58	87,74	87,58	96,02	5,29	8,30	7,33	93,79	77,36
**IL6**	1043,17	1060,36	1164,58	841,18	851,56	915,87	899,32	903,24	898,23	953,05	110,08
